# Neuroendocrine carcinoma of the endometrium: a retrospective analysis of data from a single center

**DOI:** 10.1186/s12885-024-12393-5

**Published:** 2024-05-24

**Authors:** Xueyan Liu, Yanpeng Tian, Shuping Yan, Hanlin Fu, Lulu Si, Tianjiao Lai, Meng Mao, Qian Wang, Jing Bai, Heli Li, Ruixia Guo

**Affiliations:** 1 Department of Gynecology, The First Affiliated Hospital of Zhengzhou University, No. 1, Jianshe East Road, Erqi District, Zhengzhou, Henan 450052 PR China; 2 Department of Pathology, The First Affiliated Hospital of Zhengzhou University, Zhengzhou, Henan 450052 PR China; 3 Department of Ultrasonography, The First Affiliated Hospital of Zhengzhou University, Zhengzhou, Henan 450052 PR China

**Keywords:** Neuroendocrine carcinoma, Endometrial carcinomas, Clinicopathological characteristics, Therapy, Prognosis

## Abstract

**Background:**

Neuroendocrine carcinoma (NEC) originating from the endometrium is rare, and there is limited knowledge regarding its diagnosis and optimal management. In this study, we present our experience with 11 patients with endometrial NEC, aiming to provide guidance for clinical practice.

**Methods:**

We retrospectively collected the clinical, pathological, and treatment data of 11 patients with endometrial NEC who were treated at the First Affiliated Hospital of Zhengzhou University from January 2011 to July 2023. The clinicopathological characteristics, treatment and prognosis of these patients were analyzed.

**Results:**

The median age of the patients was 55.0 (39.0–64.0) years, and the median tumor size was 40.0 (33.0–60.0) mm. Irregular vaginal bleeding was the most common symptom observed in 10 out of 11 patients, while metabolic syndrome occurred in only 2 out of 11 patients. Six out of the 11 patients were diagnosed at an early stage. Among the patients, 6 were diagnosed with endometrial NECs, while the remaining patients had a combination of endometrial NEC and other non-NEC endometrial carcinomas. All patients underwent surgery, except for one who received only chemotherapy due to multiple metastases. After surgery, adjuvant chemotherapy was administered to 5 patients, chemotherapy combined with radiotherapy was given to 3 patients, and 2 patients did not receive any adjuvant therapy. A total of 10 patients completed the follow-up, with a median follow-up time of 51.0 (14.3–81.0) months. Unfortunately, 2 patients died from the disease.

**Conclusion:**

NECs originating from the endometrium might not be affected by metabolic disorders. Preoperative diagnosis of these tumors was challenging. The primary approach for managing endometrial NEC can be multimodal treatment centered around surgery.

## Introduction

Endometrial cancer is one of the most common cancers in females worldwide and accounts for 7% of new cancer diagnoses and 4% of cancer deaths [1, 2]. Although the most common subtypes of endometrial cancer are endometrioid and serous carcinomas, some rare pathological types have been identified in recent years. Endometrial neuroendocrine carcinoma (NEC) is a rare type of endometrial cancer that originates from endocrine cells in the neural ectoderm, neural crest, and neural endoderm [3] and is characterized by high invasive and metastatic potential. Endometrial NEC, also known as endometrial high-grade neuroendocrine neoplasm (NEN), accounts for less than 1% of all endometrial cancers [4]. NENs can occur in various organs and are frequently observed in the gastrointestinal tract (62–70%), followed by the lung (25%) [3, 5]. The World Health Organization (WHO) proposed a comprehensive classification system for NENs based on the degree of cell differentiation, aiming to standardize their classification. In summary, NENs are divided into two groups: low-grade NENs and high-grade NENs. The former includes neuroendocrine tumors (NETs), such as typical carcinoid and atypical carcinoid tumors, which are rarely observed in the endometrium. The latter comprises highly aggressive neoplasms known as neuroendocrine carcinomas, which include small-cell neuroendocrine carcinomas and large-cell neuroendocrine carcinomas [6].

Endometrial NEC is generally considered to be more aggressive than other types of endometrial carcinoma [7]. A previous retrospective study from the National Cancer Database (NCDB) showed that endometrial NEC patients had a worse prognosis than endometrioid endometrial carcinoma patients, and the hazard ratio (HR) of death was 2.32 (95% CI, 1.88–2.88) [8]. In addition, large-cell neuroendocrine carcinoma has been found to have a greater risk of distant metastasis than small-cell neuroendocrine carcinoma [4]. However, due to its rarity, there are currently no guidelines or consensuses for the management of endometrial NEC. Although two large studies have summarized the clinicopathologic characteristics and prognosis of endometrial NEC using the SEER database and the National Cancer Database (NCDB) [4, 8], additional cases are needed to summarize the therapy and prognosis of endometrial NEC.

In this retrospective study, we summarized the clinicopathologic characteristics and prognoses of 11 patients with endometrial NEC from a single center. This study aimed to provide valuable insights that may guide future research efforts and clinical decision-making for patients with endometrial NEC by enhancing our understanding of the clinical features, histopathological characteristics, and treatment outcomes of endometrial NEC.

## Materials and methods

### Ethical approval

Ethical approval was obtained from the Ethics Committee of The First Affiliated Hospital of Zhengzhou University (2023-KY-0782-002). All patients and/or their families provided signed informed consent.

### Study design

This retrospective study was conducted at the Department of Gynecology, The First Affiliated Hospital of Zhengzhou University. Patients diagnosed with endometrial NEC between January 2011 and July 2023 were included. The inclusion criteria were as follows: (1) diagnosis was confirmed by histopathological examination; (2) patients with complete clinical information via the electronic medical record system; The exclusion criteria were as follows: (1) patients with other malignant tumors; (2) patients with a history of other malignancies prior to the diagnosis of endometrial NECs, and (3) patients who refused treatment for various reasons.

The study followed the Strengthening the Reporting of Observational Studies in Epidemiology (STROBE) Statement and the Reporting of studies Conducted using Observational Routinely-collected health Data (RECORD) Statement, validated by the Enhancing the Quality and Transparency Of health Research (EQUATOR) network.

### Data collection

Demographic data, International Federation of Gynecology and Obstetrics (FIGO) stage, histopathology, imaging, tumor marker levels, and treatment methods were collected from the electronic medical records system.

Patients diagnosed with metabolic syndrome (MetS) needed to meet three or more of the following conditions: (1) overweight and/or obese status (body mass index (BMI) ≥ 25.0 kg/m^2)^; (2) hyperglycemia status (fasting blood glucose ≥ 6.1 mmol/L and/or 2-hour postprandial blood glucose ≥ 7.8 mmol/L or a diagnosis of diabetes with ongoing treatment); (3) hypertension (blood pressure ≥ 140/90 mmHg or a diagnosis of hypertension with ongoing treatment); and (4) dyslipidemia (fasting triglycerides ≥ 1.69 mmol/L and/or HDLC < 1.0 mmol/L for females).

The diagnosis of NEC in the uterus was based on the WHO criteria for neuroendocrine tumors. The classification of the FIGO stage for endometrial cancer was determined using the 2008 FIGO clinical staging system [9]. Surgically resected or biopsy specimens were collected and examined by experienced pathologists. Pathology was confirmed by two expert pathologists from a single center. Patients who were initially diagnosed elsewhere underwent a histopathologic consultation. Mixed neuroendocrine carcinoma was defined as a tumor containing both neuroendocrine carcinoma and non-neuroendocrine carcinoma components [10]. Histologic type, lymph node metastasis status, and immunohistochemical data were also collected.

The diagnosis of endometrial NECs was made according to the WHO criteria for neuroendocrine tumors of the uterus. Briefly, small cell neuroendocrine carcinoma was recognized by features such as pulmonary small cell carcinoma, including tumor cells with scant cytoplasm, a “salt and pepper chromatin pattern,” and nuclear molding. Large cell neuroendocrine carcinoma was recognized by the following features: (1) the presence of polygonal cells with abundant cytoplasm and prominent nucleoli, (2) a neuroendocrine growth pattern, such as organoid nesting or trabecular or cord-like growth, and (3) the expression of at least one neuroendocrine immunomarker (chromogranin, synaptophysin, or CD56) in > 10% of tumor cells [11].

### Hematoxylin and eosin (HE) and immunohistochemistry (IHC)

All the samples were fixed in 10% neutral formalin and subsequently embedded in paraffin. Sections (4 μm) were stained with hematoxylin and eosin. Then, the paraffin-embedded blocks were cut into 4 μm slices. IHC staining was performed using an automated slide staining system (BenchMark Ultra, Roche) and an UltraView universal DAB detection kit. The IHC staining results were evaluated by pathologists experienced in gynecological pathology. The immunohistochemical markers used were as follows: synaptophysin (Syn), chromogranin A (CgA), neural cell adhesion molecule (CD56), NSE, AE1/AE3, cytokeratin 8/18 (CK8/18), CD10, estrogen receptor (ER), progesterone receptor (PR), P53, cyclin-dependent kinase-4 (P16), cytokeratin-7 (CK7), and paired box gene 8 (PAX8), as well as Ki-67. The antibody information is listed in Table 1.

**Table 1:** List of antibodies

Antibody	Manufacture
Anti-ER antibody	Roche, Germany
Anti-PR antibody	Roche, Germany
Anti-CD56 antibody	Jie Hao, China
Anti-CK7 antibody	Zhongshan Goldenbridge, China
Anti-Syn antibody	Maixin Biotech, China
Anti-PAX8 antibody	Zhongshan Goldenbridge, China
Anti-AE1/AE3 antibody	Maixin Biotech, China
Anti-CgA antibody	Jie Hao, China
Anti-P53 antibody	Zhongshan Goldenbridge, China
Anti-P16 antibody	Roche, Germany
Anti-Ki−67 antibody	Gene Tech, China

The primary endpoint was overall survival (OS), which was defined as the time from diagnosis to the last follow-up visit or death. The secondary endpoint was progression-free survival (PFS), which was defined as the time from diagnosis to the first recurrence, progression of disease or death. The surveillance of NEC included physical examination, serum tumor marker levels and abdominal-pelvic and thoracic imaging every 3 months for 2 years, every 6 months for 3 years and yearly thereafter. Patients were followed up via telephone and outpatient services. The last follow-up time was August 2023.

### Statistical analysis

The statistical analysis was conducted using SPSS 26.0 software. Continuous variables are presented as medians (ranges), while categorical data are reported as counts and percentages. K‒M curves were generated to estimate progression-free survival (PFS) and overall survival (OS).

## Results

A total of 11 patients were included in this study; 7 patients were initially diagnosed at our hospital, and 4 patients were diagnosed at other hospitals. The median age was 55.0 (39.0–64.0) years. Among these patients, 7 out of 11 were menopausal, and only 1 patient had no history of pregnancy. Irregular vaginal bleeding (10 out of 11) was the most common symptom and included postmenopausal bleeding (6 out of 11) and abnormal uterine bleeding (4 out of 11). Additionally, 4 out of 11 patients had hyperglycemia, and 2 out of 11 had a BMI ≥ 25 kg/m^2^. Furthermore, 3 out of 11 patients had hypertension, and 3 out of 11 had dyslipidemia. Metabolic syndrome was observed in 2 out of 11 patients. All patients underwent physical examination prior to treatment; 6 patients showed no abnormal signs, and 5 patients showed uterine enlargement. According to the FIGO staging system, 6 patients were at an early stage of the disease (stage I-II), while 5 patients were at an advanced stage (stage III-IV). Lymph node metastasis occurred in 5 patients. The details are shown in Table 2.

**Table 2:** Patient characteristic of 11 cases of neuroendocrine carcinoma of the endometrium

NO.	Age	Menopause	Symptom	Comorbidity	Reproductive history	FIGO stage	Tumor size(mm)	Lymphatic metastasis	Surgery	RT	CT	Follow-up
1	54	Yes	Postmenopausal bleeding	Hypertension, Hyperglycemia, Dyslipidemia	G2P2	IIIC2	55	Yes	MRH + BSO + LND	Yes	TP	90 mon, NED
2	28	No	Abnormal uterine bleeding	No	G0	IA	42	No	EH + LND	No	TP	78 mon, NED
3	39	No	Abnormal uterine bleeding	No	G3P2	IA	33	No	EH + BSO + LND	No	TP	37 mo, NED
4	61	Yes	Postmenopausal bleeding	No	G4P4	IIIC2	24	Yes	EH + BSO + LND	No	TP	105 mo, NED
5	67	Yes	Postmenopausal bleeding	No	G3P3	IA	33	No	EH + BSO + LND	No	EP	59 mo, NED
6	55	No	Abnormal uterine bleeding	Hyperglycemia, Overweight	G3P3	IA	60	No	EH + BSO + LND	Yes	EP	51 mo, NED
7	77	Yes	Postmenopausal bleeding	Hyperglycemia	G4P4	IA	40	No	EH + BSO	No	No	NA
8	56	Yes	Postmenopausal bleeding	Hypertension	G3P3	IIIC2	74	Yes	EH + BSO + LND	No	No	15 mo, DOD
9	64	Yes	Vaginal drainage	Hypertension, Hyperglycemia, Dyslipidemia, Overweight	G5P4	IA	34	No	EH + BSO + LND	Yes	TP	51 mo, NED
10	48	Yes	Postmenopausal bleeding	No	G1P1	IVB	68	Yes	EH + BSO + LND	No	TP	12 mo, DOD
11	34	No	Abnormal uterine bleeding	Dyslipidemia	G4P2	IVB	25	Yes	No	No	EP	1 mo, NED

MRH: Modified radical hysterectomy; EH: Extrafascial hysterectomy; BSO: Bilateral salpingo-oophorectomy; LND: Lymph node dissection; CT: Chemotherapy; RT: Radiotherapy; TP: Taxol + Platinum; PE: Etoposide + Platinum; DOD: Died of disease; NA: Not available; NED: No evidence of disease; mo: Month

The median tumor size was 40.0 (33.0–60.0) mm. Among these lesions, the majority were confined (7 out of 11). Four lesions were located in the uterine fundus, and 3 lesions were located in the lower uterine cavity. The remaining 3 patients had extensive lesions that involved the entire uterine cavity. The tumor markers CA125 or neuron-specific enolase (NSE) were elevated in 5 patients at the time of diagnosis. Elevated CA125 levels were observed in 4 out of 10 patients, while elevated NSE levels were observed in 3 out of 5 patients. All patients underwent pelvic ultrasound examination, which revealed an ill-defined border in 8 out of 11 patients, heterogeneous echogenicity in 7 out of 9 patients, and abundant blood flow signals in 8 out of 11 patients. Additionally, 4 patients underwent pelvic MRI. On T1WI, 1 out of 4 tumors had intermediate signal intensity, and 3 had low signal intensity. All 4 patients had high signal intensity on T2WI. Furthermore, all 4 tumors exhibited intense low signal intensity on apparent diffusion coefficient (ADC) maps and intense high signal intensity on diffusion weighted imaging (DWI) throughout the mass. The results of dynamic contrast enhancement scanning in 4 patients showed a washout/plateau pattern, as detailed in Table 3.

**Table 3:** Parameter about imaging and ecsomatics prior to treatment

NO.	Ultrasound			MRI						CA125 (U/ml)	CA153 (U/ml)	CA199 (U/ml)	HE4 (pmol/l)	CA724 (U/ml)	CEA (ng/ml)	AFP (ng/ml)	CYFRA21-1 (ng/ml)	NSE (ng/ml)
	Border	Echogenicity	CDFI	Border	T_1_WI	T_2_WI	ADC	DWI	Dynamic contrast enhancement pattern									
1	Ill-defined	Heterogeneous	2–3	Ill-defined	Low	High	Low	High	Washout/plateau pattern	-	NA	-	NA	NA	-	-	NA	NA
2	Ill-defined	Homogeneous	3	NA						-	NA	-	-	NA	-	-	NA	NA
3	Well-defined	Heterogeneous	2–3	NA						-	-	-	NA	-	-	-	-	-
4	Ill-defined	Homogeneous	3	Ill-defined	Iso	High	Low	High	Washout/plateau pattern	NA	NA	NA	NA	NA	NA	NA	NA	NA
5	Well-defined	NA	1	NA						-	NA	-	-	NA	-	-	NA	NA
6	Ill-defined	NA	2–3	NA						37.26	NA	-	-	NA	-	-	NA	NA
7	Ill-defined	Heterogeneous	1	NA						-	NA	-	164	NA	-	-	NA	NA
8	Well-defined	Heterogeneous	2–3	Ill-defined	Low	High	Low	High	Washout/plateau pattern	40.65	-	-	-	-	NA	-	-	152.8
9	Ill-defined	Heterogeneous	0	Ill-defined	Low	High	Low	High	Washout/plateau pattern	40.64	-	-	NA	-	-	-	-	-
10	Ill-defined	Heterogeneous	2–3	NA						-	-	-	-	-	-	-	-	105
11	Ill-defined	Heterogeneous	2–3	NA						348	-	-	NA	-	-	-	19.8	459

-: negative; NA: not available; CDFI: Color Doppler flow imaging

**Fig. 1:**
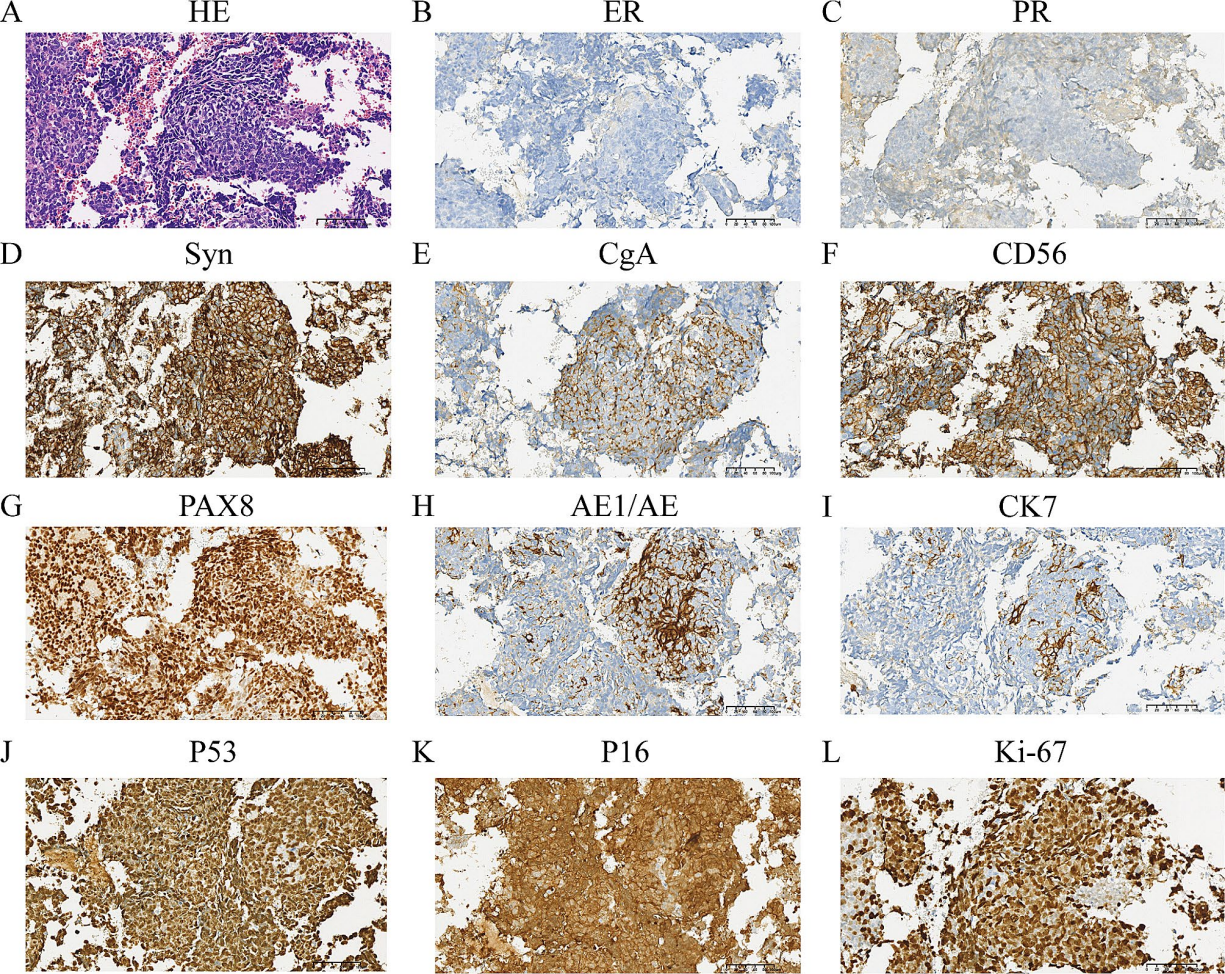
HE staining and immunohistochemical staining results for Patient 10. HE staining and immunohistochemical staining of Patient 5. ER and PR were negative. (**A**) HE staining. (**B**) ER. (**C**) PR. (**D**) Syn. (**E**) CgA. (**F**) CD56. (**G**) PAX8. (**H**) AE1/AE3. (**I**) CK7. (**J**) P53. (**K**) P16. (**L**) Ki-67. Notes: ER, estrogen receptor; PR, progesterone receptor; GATA3, GATA binding protein 3; CD, cluster of differentiation; TTF1, transcription termination Factor 1; PAX8, paired-box gene 8

Of the 11 patients, 6 patients were diagnosed with only endometrial NEC, while 5 patients were diagnosed with mixed neuroendocrine carcinoma. Among the mixed neuroendocrine carcinoma patients, 3 had endometrioid carcinoma, 1 had combined sarcoma, and 1 had combined undifferentiated carcinoma. Lymphovascular space invasion occurred in 4/8 of the patients. The depth of myometrium invasion was more than 50% in 3/9 patients, all of whom were at an advanced stage. All tumors were positive for one or more neuroendocrine markers, including Syn, CgA, CD56, and NSE. However, 8 out of the 11 tumors were negative for both ER and PR. Immunohistochemical examinations of the specimens revealed positive reactions for synaptophysin in 10 out of 10 patients, CgA in 3 out of 7 patients, CD56 in all 11 patients, and NSE in 2 out of 2 patients. Additionally, 100% of the patients were positive for P53 (7 out of 7 patients), P16 (7 out of 7 patients), AE1/AE3 (5 out of 5 patients), and CD8/18 (2 out of 2 patients). The Ki-67 labeling index was assessed in 10 patients and ranged from 30 to 80%, with a median value of 75%. Lymph node metastasis occurred in 5 out of the 10 patients. For more details, please refer to Table 4. Representative IHC staining results from Patient 10 are shown in Fig. 1.

**Table 4:** Immunohistochemical staining results in neuroendocrine carcinoma of the endometrium

NO.	Histopathologic type	Syn	CD56	CgA	NSE	ER	PR	CD10	AE1/AE3	P16	Ki-67	P53	CD8/18	EMA	CK7	PAX8
1	SCNC	+	+	NA	NA	-	-	-	NA	NA	70%+	+	NA	+	NA	NA
2	SCNC	+	+	-	+	-	-	-	NA	+	60%+	NA	NA	-	-	NA
3	SCNC + UC	+	+	-	NA	-	-	NA	NA	NA	80%+	+	NA	NA	+	-
4	SCNC + EC	+	+	NA	+	-	-	-	NA	NA	70%+	NA	NA	+/-	NA	NA
5	NEC + SC	+	+	NA	NA	-	+	-	+	+	80%+	+	+	+	+	NA
6	LCNC + EC	+	+	+	NA	+	+	+	+	+	NA	NA	NA	NA	+	NA
7	NEC	+	+	-	NA	-	-	+	+	+	80%+	+	+	NA	NA	NA
8	NEC	+	+	+	NA	-	-	NA	+	+	70%+	+	NA	NA	-	NA
9	SCNC + EC	NA	+	-	NA	+	-	+	NA	+	30%+	+	NA	NA	NA	NA
10	SCNS + LCNC	+	+	+	NA	-	-	+	+	+	80%+	+	NA	NA	+	+
11	SCNC	+	+	NA	NA	-	-	NA	NA	NA	80%+	NA	NA	NA	NA	NA

SCNC: small cell neuroendocrine carcinoma; LCNC: large cell neuroendocrine carcinoma; EC: Endometrioid carcinoma; UC: Undifferentiated carcinoma; SC: sarcoma; NEC: neuroendocrine carcinoma; +: positive; -: negative; NA: not available

**Fig. 2:**
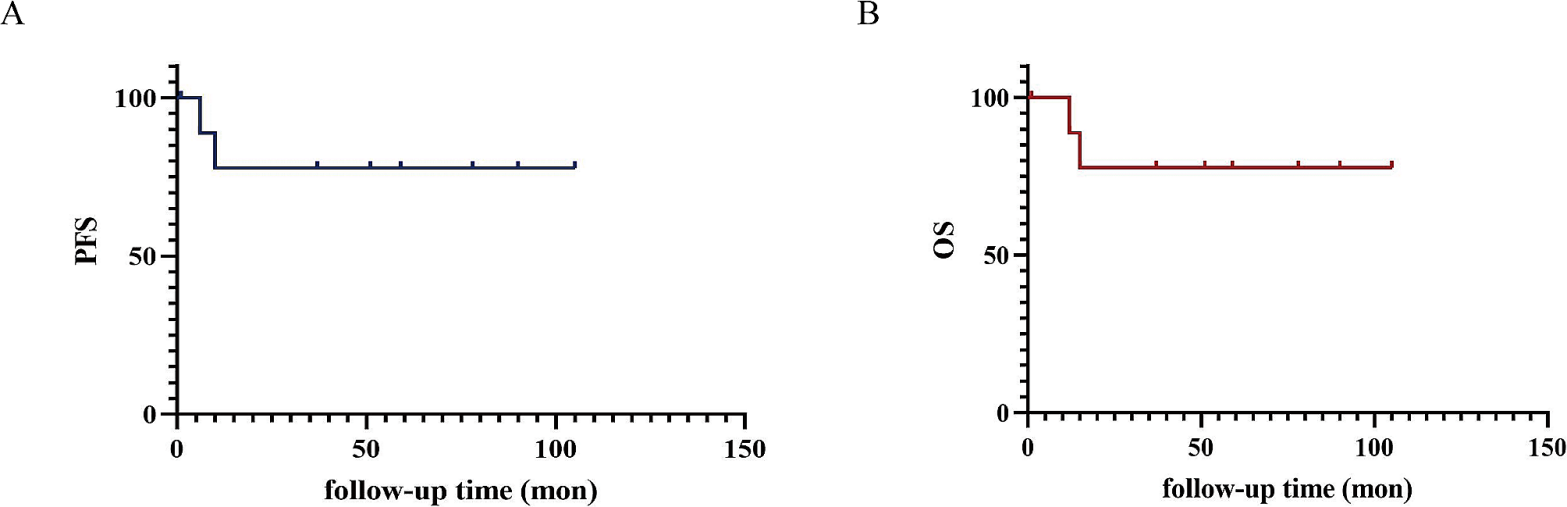
Survival curves of 10 patients with endometrial NECs (**A**, PFS; **B**, OS)

The initial treatments administered for each patient are shown in Table 1. One patient received chemotherapy only due to multiple metastases, while the remaining 10 patients underwent surgery. Eight of those patients underwent comprehensive staging surgery, involving hysterectomy, bilateral salpingo-oophorectomy, and lymph node dissection. One patient underwent hysterectomy and lymph node dissection at a young age, and another patient underwent hysterectomy and bilateral salpingo-oophorectomy due to the presence of comorbid disease and related anesthesia problems. After surgery, 5 patients received adjuvant chemotherapy, and 3 patients received chemotherapy and radiotherapy. In addition, 2 patients did not receive any adjuvant therapy—including one patient with stage IA disease and one with stage 3 disease—who refused to return to the hospital for adjunctive therapy due to financial burdens, although the doctor had adequately informed themselves of the need for adjuvant radiotherapy and chemotherapy. The most common chemotherapy regimen for those who received chemotherapy was Taxol + Platinum (6 out of 9 patients), followed by Etoposide + Platinum (3 out of 9 patients). Three patients received adjuvant radiotherapy after surgery—1 with stage IIIC2 disease and 2 with stage IA disease. All of the 3 patients did not experience recurrence until August 2023. Of the patients who completed treatment, all achieved complete remission. Up to August 2023, 10 patients completed follow-up, for a median follow-up time of 51.0 (14.3–81.0) months. The 5-year overall survival (OS) and 5-year progression-free survival (PFS) rates were both 77.8%. Two patients with advanced disease experienced tumor recurrence and died within 2 years after diagnosis. One patient who subsequently died of rapidly progressive disease underwent renal failure after recurrence and only received conservative herbal remedies; the other patient received further treatment at an outside hospital after recurrence, and we could not obtain additional detailed information related to the treatment regime. Further details can be found in Table 4; Fig. 2.

## Discussion

There have been few reports of endometrial NECs in China to date, which makes managing this condition a great challenge for general clinicians due to their limited clinical experience. To the best of our knowledge, this study presents the largest reported case series on the Chinese population in a single center involving 11 patients with endometrial NECs. We explored the clinicopathological features, imaging features, serum tumor marker changes and prognosis of the Chinese population with endometrial NECs thoroughly; these characteristics have rarely been reported in previous studies. In addition, we reported the detailed treatment methods, including surgical procedures, chemotherapeutic regimens, and radiotherapy, based on Chinese experience. To our knowledge, endometrial NECs might present different clinical characteristics than endometrial non-NECs reported previously.

The association between endometrial cancer and metabolic disorders has been well established and is particularly true for type I endometrial cancer. In a retrospective study conducted by Yang X et al., 30.2% of the 506 patients with endometrial carcinoma were found to be associated with metabolic syndrome [12]. Additionally, 39.7% had hyperglycemia, 56.3% were overweight, 41.3% had hypertension, and 49.6% had dyslipidemia. Besides, 44.1% of patients with mixed endometrial epithelial carcinoma had a BMI over 30 kg/m^2^, as shown by Christina Pappa [1]. In our study, the proportion of individuals with metabolic disorders or metabolic syndrome was lower than what was reported in the aforementioned study, especially in terms of dyslipidemia and overweight. This finding suggested that the relationship between endometrial NEC and metabolic disorders or metabolic syndrome may not be as significant as that observed in endometrial non-NEC patients. Similarly, a case series of small cell endometrial cancer conducted by Koo Y-J et al. was consistent with our study, showing that 1 out of 6 patients were overweight [13]. Certainly, these differences, compared to those in endometrial non-NEC patients, may be influenced by the small sample size of our study. The association with metabolic disease could also be influenced by other confounding factors, including age and race, all of which need to be verified in larger studies. Furthermore, it should be acknowledged that there are varying definitions of metabolic syndrome in different studies, while information such as waist circumference data was not recorded in our study, which limited the analysis of different definitions of MetS. However, further exploration is needed to fully understand the association between metabolic diseases and endometrial NECs, and the above difference might be an entry point for future molecular studies to some extent.

According to previous studies, most patients with endometrial NEC are diagnosed at an advanced FIGO stage due to the high potential for invasion and metastasis [4, 8], while more than 90% of endometrial non-NECs were diagnosed at an early stage [14]. A retrospective study by J Zhang examined 170 patients with endometrial NECs from the SEER database, 83.5% of whom were initially diagnosed at Stages III-IV [4]. Similarly, Matsumoto H et al. analyzed 42 patients with endometrial NEC from the Kansai Clinical Oncology Group (KCOG) and reported that 69% of them presented with advanced FIGO stage disease during their initial hospital visit [15]. In our study, 45.5% of patients presented with FIGO stage III or IV disease, indicating a lower percentage of advanced-stage patients than in the aforementioned studies. Although our study involved only 11 patients, this discrepancy should not be ignored. This difference could be attributed to socioeconomic development, as the patients included in the previous cohorts were studied more than a decade ago. As highlighted by Schlechtweg K et al., patients with endometrial NEC were less likely to receive a diagnosis prior to 2008 and present with stage I disease than were those with endometrial non-NEC [8]. The development of disease screening and diagnosis has progressed rapidly alongside economic and technological advancements. Furthermore, the growing awareness and demand for health management among the public have encouraged timely visits to hospitals, leading to early diagnosis.

An efficient screening method could help with triage and follow-up. In this study, ultrasound and MRI were the two main radiologic examinations used. Lesions observed via ultrasound showed poorly defined boundaries and abundant blood flow signals, similar to common endometrial malignancies. Pelvic MRI had imaging parameters similar to those of a previous study, including an ill-defined border between the endometrium and myometrium, diffuse infiltration and high signal intensity on T2WI, and abnormal high signal intensity throughout the tumor as shown on DWI. These findings were consistent with those of other endometrial non-NEC and malignant tumors of the uterine corpus that invade the endometrium [16]. In summary, a definitive diagnosis was difficult based on preoperative imaging findings alone. However, it cannot be denied that the tumor size in this study tended to be large, with a median size of 4.0 cm. Although most previously published studies on endometrial NEC did not provide the maximum tumor dimensions, large bulky tumors have been described. Similarly, a large cohort study involving 25 patients with endometrial NEC from a previous study reported a median tumor size of 6 cm [11]. Akgor U et al. reported a case of small-cell NEC with the largest size tumor in the literature, measuring 18 cm in diameter [17]. This study revealed that elevated CA125 and NSE levels were the most common tumor markers, which is consistent with the findings of other reports [18]. CA125 is well known for its role in ovarian epithelial cancer and endometrial carcinoma. NSE, on the other hand, is a highly specific marker for neurons and peripheral neuroendocrine cells and is typically found in specific tissues under normal conditions. However, elevated levels of NSE can indicate malignant proliferation and are currently an essential tumor marker for the diagnosis, prognosis, and follow-up of small-cell lung carcinoma (SCLC). Therefore, screening for NSE should be considered in patients with large bulky malignancies of the endometrium.

Adverse risk factors are important for adjuvant therapy for endometrial carcinoma confined to the uterus. As suggested by the National Comprehensive Cancer Network (NCCN) clinical practice guidelines, adverse intrauterine pathologic risk factors include high-grade tumors, deep myometrial invasion (and consequently more advanced stage), lymphovascular invasion (LVSI) (especially extensive), and serous or clear cell carcinoma histologies [19]. In our study, lymphovascular space invasion occurred in 4/8 of the patients, and the depth of myometrial invasion was more than 50% in 3/9 of the patients, all of whom were at an advanced stage. Regrettably, we were unable to assess specific high-risk factors because of the limited sample size. Adverse high-risk factors for endometrial non-NECs suggested by the National Comprehensive Cancer Network (NCCN) are still referable due to the lack of additional high-quality evidence. In addition, MMR or MSI testing is essential for guiding treatment and survival in patients with endometrial carcinoma. Future studies investigating immune checkpoint therapy for this rare tumor type might also support the use of endometrial NECs, which are often not included in clinical trials. However, neither MMR nor MSI testing was performed in this study for various reasons. This was a limitation of our study. More sufficient evidence about MMR and MSI testing in patients with endometrial NECs is needed in follow-up clinical work.

There are no established guidelines or consensuses for the treatment of endometrial NEC due to a lack of high-quality research evidence. The optimal treatment approach is still being investigated. The Society of Gynecologic Oncology (SGO) recommends multimodal treatment strategies for endometrial NEC, which include surgery, chemotherapy, and radiotherapy [20]. Surgery is considered the best curative option, and complete resection is crucial for improving survival, as previously suggested [15]. We herein adopted multimodal treatment based on surgery, and all patients who completed the treatment achieved complete remission. However, these criteria were not specific for patients who experienced recurrence in our study, where two patients with advanced disease experienced recurrence and died within 2 years after diagnosis. One patient who subsequently died of rapidly progressive disease underwent renal failure after recurrence and only received conservative herbal remedies; the other patient received further treatment at an outside hospital after recurrence, and we could not obtain additional detailed information related to the treatment regime. In addition, there is currently no consensus regarding chemotherapy regimens. Most studies recommend a combination of etoposide and platinum-based treatment for small cell lung cancer [17, 21]. Additionally, some reports have utilized a combination of Taxol and platinum-based agents, which aligns with the treatment approach for endometrial non-NEC [22, 23]. In our study, 33.3% of the patients received a combination of etoposide and platinum, while 66.7% of the patients received a combination of Taxol and platinum. Owing to the small sample size, this study did not include a comparison of patient prognosis among patients with different FIGO stages or treatment methods. In conclusion, it remains unclear whether endometrial NEC should be managed based on the treatment experience of patients with endometrial non-NEC or small cell lung cancer. However, further exploration via additional case reviews and clinical trials is needed.

The 5-year overall survival (OS) and 5-year progression-free survival (PFS) rates in this study were both 77.8%, which appear to be better than those of previously reported endometrial NECs and mixed endometrial epithelial carcinoma [4, 8, 24]. Moreover, as noted above, survival was worse for endometrial NEC patients than for endometrioid endometrial carcinoma patients at all stages [8]. However, the American Cancer Society and the National Cancer Institute presented a 5-year relative survival rate of 81% for all stages of endometrial carcinoma in 2022 [25], which is similar to the rate observed in our study for endometrial NEC. This finding is inconsistent with the previous conclusion that endometrial NECs are more aggressive than endometrioid carcinomas [8]. A possible explanation may be related to the higher ratio percentage of early-stage patients in our study. Additionally, ethnic differences may also have some impact on the prognosis. However, differences in treatment methods, especially chemotherapy regimens, may also affect patient survival. Immune checkpoint blockade therapy was confirmed to have durable clinical benefits for previously treated or metastatic advanced noncolorectal MSI-H/dMMR cancers, most of which were endometrial cancer [26–28]. Furthermore, Cady E showed that MMR protein expression abnormalities occurred in 8/18 endometrial NECs, which was higher than that observed in endometrial cancer (approximately 17%) [11, 26, 29]. In summary, immune checkpoint blockade therapy for endometrial NEC is promising, although there is insufficient evidence to confirm this conclusion.

In this study, we retrospectively analyzed the clinicopathological characteristics and prognosis of 11 endometrial NEC patients. These findings suggested that endometrial NEC might be more aggressive than endometrial endometrioid carcinoma. This study helps to characterize endometrial NEC. Nonetheless, our study has several drawbacks, including its retrospective design, small sample size, and single-center design. Specifically, the study group was not compared to any control group, and we were not able to compare treatment modalities due to the limited cases. More prospective studies should be conducted with larger sample sizes and multicenter designs.

## Data Availability

All data generated or analyzed in this study are included in this published article.
